# Excellence in cell signaling research recognized with major new award

**DOI:** 10.1186/1478-811X-11-17

**Published:** 2013-03-04

**Authors:** Stephan M Feller

**Affiliations:** 1 Biological Systems Architecture Group, Weatherall Institute of Molecular Medicine, University of Oxford, Oxford OX3 9DS, UK

## Abstract

The newly installed Life Sciences Breakthrough Prize (http://www.breakthroughprizeinlifesciences.org/), which comes with more than double the financial reward of the Nobel Prize, has been awarded to several world-leaders in the field of cancer-related cell signaling and therapy research: Lewis C. Cantley (PI3 kinase), Hans Clevers (Wnt signaling), Charles L. Sawyers (signaling-targeted cancer therapy), Bert Vogelstein (colorectal cancer signaling) and Robert Weinberg (Ras & other cancer-relevant genes). They have all made remarkable contributions to our understanding of cell communication and malignancies over the last decades. Needless to say that virtually all other awardees of the 11 scientists honored in 2013 have also, in one way or another, touched upon signaling molecules, highlighting the fundamental interdisciplinarity and significance of signal transduction for living cells in general. For example, Shinya Yamanaka’s exciting work was built on the four transcriptional signaling proteins, Oct3/4, Sox2, Klf4 and c-Myc.

## 

Importantly, the soon to come public lectures of the awardees will be made available on the *Life Sciences Breakthrough Prize* website and can thus provide a resource to possibly inspire some young students to read more about this exciting research realm.

Additional inspiration can come from a couple of outstanding books, which I would like to briefly highlight here:

*Natural Obsessions* by Natalie Angier, originally published in 1988, which highlights some of Bob Weinberg’s work, is now available again in a new edition.

*The Emperor of all Maladies* by Siddhartha Mukherjee (*Pulitzer Prize* winner of 2011 in the category General Non-fiction) is a fantastic time travel through the history of cancer therapies.

Many more exciting and dramatic stories of breakthrough signaling discoveries, however, still remain to be captured for general audiences. Maybe the *Life Sciences Breakthrough Prize* could inspire a few of the best science writers to preserve more of these stories in coming years.

On a slightly less enthusiastic note, it is not entirely clear to me how much this massive new prize as such will actually accomplish with respect to one of its supposed major aims, that is “…generating excitement about the pursuit of science as a career.” Major financial rewards are clearly far out of reach for the vast majority of scientists, no matter how excellent, so a career as stockbroker or fund manager will remain much more enticing to young wannabe multi-millionaires.

Furthermore, bridging the currently existing communication gap between those ingenious researchers in their ‘ivory towers’ and the common public, including putative future science supertalents, might be better accomplished by other means than showering a few fortunate individuals with a tiny sprinkle of the accumulated multi-billion ‘silicone valley’ profits, which have been - at least to some significant degree - accumulated by very clever tax evasion.

Clearly, open access publishing is one of these means. Publically funded scientific results must be made immediately accessible to all.

Moreover, we need to train young scientists to be able to communicate their exciting findings in a language that is much more accessible to all reasonably educated lay persons and certainly much more coherent than the secret codes and TLAs currently used.

Having gifted public science writers ‘in residence’ much more frequently embedded in world-leading science institutes might also be a good investment.

Last not least, science teaching in many public schools is vastly underfunded and an investment in those young and very eager minds, albeit being clearly less glamorous than competing with the *Nobel Prize*, could potentially be more fertile than spending those millions on much more seasoned members of our species.

So will this new award have real impact on the pace of scientific discovery and hence be more than just a clever PR-stunt to take some heat off a few multi-billion dollar corporations that have come under increasing public scrutiny in recent years? Only time will tell.

